# It Ain’t Over Till It's Over: The Triple Threat of COVID-19, TB, and HIV

**DOI:** 10.4269/ajtmh.20-1089

**Published:** 2020-08-31

**Authors:** Alexander W. Kay, Tara E. Ness, Leonardo Martinez, Anna M. Mandalakas

**Affiliations:** 1 Baylor College of Medicine Children’s Foundation Eswatini, Mbabane, Eswatini;; 2 Baylor College of Medicine and Texas Children’s Hospital, Houston, Texas;; 3 Stanford University, Stanford, California

As the SARS-CoV-2 outbreak rapidly spread to pandemic proportions, the global tuberculosis (TB) and HIV care communities voiced increasing concerns. Can we contain the inevitable disruption of routine TB and HIV services and the resultant detrimental impacts to patient care and disease control? Tuberculosis alone kills 4,000 people each day and 1.5 million people each year. The impact of TB is greatest in settings with high burdens of both TB and HIV, where a third of people living with HIV die from TB. In 2018, an estimated 10 million people developed TB, nearly half a million drug-resistant TB. Although an estimated 58 million lives were saved through TB diagnosis and treatment between 2000 and 2018,^1^ the current SARS-CoV-2 pandemic threatens to reverse these gains by overwhelming the healthcare system, disrupting patient access to care, and spurring reallocation of resources from TB and HIV services. Furthermore, in already stressed settings with high burdens of TB and HIV infection, the risk and impact of triple infection with TB, HIV, and SARS-CoV-2 will likely be greatest.

In this issue of the AJTMH, two articles describe four patients with triple infection with drug-sensitive *Mycobacterium tuberculosis*, HIV, and SARS-CoV-2.^2,3^ Farais et al. describe two adult male patients in Brazil, both with poor antiretroviral treatment (ART) adherence and viremia. One individual had known active TB, but did not complete treatment, and the other had TB diagnosed upon presentation with COVID-19. Although these patients ultimately recovered, the authors note the challenge of delaying ARTs because of TB treatment. Rivas et al. describe another two adult males, both with uncontrolled viremia from HIV with low CD4 counts. One had TB diagnosed during hospitalization, and the other was admitted during the continuation phase of TB treatment. Whereas ART initiation was delayed because of TB treatment, management of COVID infection did not alter the overall treatment course for TB or HIV; however, none of the patients were treated with remdesivir, a drug increasingly used in the care of patients with severe SARS-CoV-2, which has a significant interaction with the TB drug rifampicin.

The authors of these case reports effectively describe the preliminary and often conflicting available data on the association between COVID-19 and clinical outcomes in patients with HIV and TB coinfection. The diversity of conclusions drawn in the literature to date, from those suggesting that HIV may have a protective effect,^2,3^ to a negligible effect,^4–6^ and to a deleterious effect,^7^ may reflect the heterogeneity of the global HIV epidemic itself. Emerging data from South Africa, a country with a generalized HIV epidemic, suggest that HIV is associated with an increased risk of COVID-19 mortality. Even in this setting, however, the impact of HIV on COVID-19 mortality is dwarfed by the impacts of noncommunicable diseases such as diabetes mellitus and hypertension, which are increased among people living with HIV.^7^ Similarly, the same risk factors that predispose individuals to TB, such as advanced age, kidney disease, diabetes, and male gender, also confer risk for severe COVID-19 disease.^8^ A South African cohort demonstrated a 1.5- to 2-fold increased risk of COVID-19 mortality associated with prior or current TB disease, respectively. However, it is difficult to separate the impact of COVID-19 from the increased risk of TB itself, and other case series have not found a similar association.^9^ These cases, and the limited literature to date, highlight the importance of better establishing the roles that HIV- and TB-specific risk factors play in COVID-19 outcomes, either independently or together.

Although comorbidities associated with HIV and TB may primarily drive COVID-19 disease severity in these populations, the disruption to health systems caused by the COVID-19 pandemic is likely to have a greater impact on the lives of people with HIV infection and TB than is coinfection with COVID-19.^10^ Preliminary data from South Korea, China, and Nigeria, all locations that have seen substantial reductions in TB over the past decade, suggest that the COVID-19 pandemic has severely disrupted TB control services. In South Korea, TB notifications decreased by 24% in 2020 compared with prior years.^11^ Similarly, in Nigeria, there were 35% and 34% decreases in the number of presumptive and active TB notifications from January to May 2020, respectively.^12^ Mathematical models predict that by 2024, deaths due to HIV and TB will increase by 10% and 20%, respectively, compared with levels predicted without a COVID-19 pandemic.^13^

There is, however, a potential opportunity to harness efforts to control COVID-19 in a way that also mitigates impacts on TB and HIV control.^14^ This may include coordinating contact-tracing efforts, which are critical to controlling outbreaks of both COVID-19 and TB. Importantly, novel technology that has been developed for COVID-19 contact tracing could be an important tool to increase efficiency of contact tracing of TB patients.^15,16^ In addition, community health workers newly designated to screen for COVID-19 may provide a workforce with experience and training compatible with HIV testing and TB screening services.

The COVID-19 pandemic has highlighted the need for all countries to invest in universal health coverage and to ensure equitable distribution of not only COVID-19 treatment and vaccines but also TB and HIV infection treatments. COVID-19 has already depleted and disrupted the global supply chain for all drugs, including TB and HIV medicines, and personal protective equipment (e.g., N95 respirators) used in a variety of illness settings. Focusing solely on COVID-19, especially in these low-resource settings, will damage the very fragile gains we have made in TB and HIV control. An approach that does not consider TB and HIV control will inevitably be less effective, and will stand as a missed opportunity to rise to the challenge of ending TB and HIV infection.
